# Construction of a prediction model for hepatocellular carcinoma based on machine learning and its prognostic characteristics

**DOI:** 10.1097/MD.0000000000044966

**Published:** 2025-10-10

**Authors:** Jiaming Wang, Tongping Shen, Shihao Wang

**Affiliations:** a School of Information Engineering, Anhui University of Chinese Medicine, Hefei, China.

**Keywords:** hepatocellular carcinoma, machine learning, prediction model, prognostic genes, SHAP, shiny, tumor mutation load

## Abstract

Hepatocellular carcinoma (HCC), as a cancer with high morbidity and mortality, urgently requires the development of a clinical prediction model with high robustness and generalizability and its prognostic study of the tumor microenvironment to provide personalized clinical treatment for patients. Key prognostic genes were screened by analyzing mRNA expression data from GTEx and The Cancer Genome Atlas (TCGA) using limma difference analysis, Cox analysis, and machine learning (ML) algorithms. TCGA database was used as a training set, and the International Cancer Genome Consortium database was used as a test set to screen the best prognostic modeling algorithms using a combination of 101 ML algorithms for training and constructing Nomo score plots based on the algorithmic risk scores as well as Shiny online prediction models. Based on shapley additive explanations analysis, drug sensitivity analysis, and immune infiltration analysis were performed on the 6 genes screened to visualize the importance of prognostic genes. HCC tumor mutation load analysis was also performed. A risk prediction model for HCC death was developed based on the RSF algorithm, with an RSF model C-index of 0.765 and AUC values of 0.978, 0.989, and 0.964 for 1-, 3-, and 5-year ROC curves for the Nomo score model, respectively. LPL, RAET1E, RNASEH2A, GTF2H4, SCML2, and PRDM12 were potential diagnostic and prognostic markers, among which SCML2 and PRDM12 were significantly correlated with multiple drugs in drug sensitivity analysis.TP53 mutations were correlated with patients’ age, chronological age, gender, histological tumor stage, T stage, and lymph node metastasis. An online HCC mortality risk prediction model was developed using the RSF algorithm. LPL, RAET1E, RNASEH2A, GTF2H4, SCML2, and PRDM12 are potential prognostic target genes, whereas TP53 mutations are associated with clinical features that may inform the development of HCC therapy.

## 1. Introduction

Primary hepatocellular carcinoma remains one of the leading causes of cancer-related deaths globally, with approximately 900,000 new cases and 830,000 deaths per year, making it the sixth most common cancer and the third most lethal cancer worldwide.^[1,2]^ Its main risk factors include hepatitis B virus and hepatitis C virus infections, alcohol consumption, smoking, and nonalcoholic fatty liver disease (NAFLD).^[3–6]^ Primary hepatocellular carcinoma is mainly categorized into hepatocellular carcinoma (HCC) and intrahepatic cholangiocarcinoma (ICC), with HCC accounting for about 75% of all cases and being the most common type.^[7]^ As HCC is usually diagnosed late, early diagnosis and prevention are crucial.^[8]^

Despite the progress made in the prevention and control of HCC through the promotion of the hepatitis B vaccine, antiviral therapy, and the development of gene editing technology, its overall survival (OS) rate is still low, which is mainly attributed to the late diagnosis and the limitations of the available treatments.^[9–12]^ Early screening and precise diagnosis of HCC have a key role in improving the prognosis; thus, establishing an efficient prediction model is particularly important. Recently, gene expression profiling has gradually become an important tool for prognostic assessment of HCC, which helps reveal the molecular mechanisms of its occurrence, development, and therapeutic response.^[13]^ Meanwhile, immune infiltration analysis provides a new perspective for HCC prognostic studies, further refining prognostic prediction by resolving the role of immune cells in the tumor microenvironment (TME).^[14–17]^ It has been shown that high levels of tumor-infiltrating CD8 + T cell expression correlate with a favorable prognosis in HCC patients. In contrast, increased macrophage infiltration suggests poorer OS and disease-free survival.^[18,19]^

Despite the advances in diagnostic and therapeutic technologies, a single genetic analysis or immune infiltration assessment can no longer meet clinical needs; therefore, combining HCC prognostic models with tumor microenvironmental prognostic analyses to optimize risk stratification and guide individualized treatments has become a key direction of current research.

In recent years, machine learning (ML), as an emerging discipline, has become an important tool for oncology research and has shown great potential for application in the field of HCC, especially providing advanced methods in data analysis, pattern recognition, and predictive modeling.^[20–24]^ While traditional prognostic models rely on limited clinical parameters, ML algorithms can integrate and process high-dimensional data to learn potential prognostic factors from training data.^[25]^ Based on the continuous development of transcriptome technology, ML has been gradually applied to the analysis of high-dimensional transcriptome data and has achieved superior research results.^[26]^ Zhang et al used SVM and other algorithms to analyze the transcriptome data of 1190 cases of HCC and 62 cases of CwoHCC, and the sensitivity, specificity, and AUC of the constructed prediction model reached 91.93%, 100%, and 0.9597 (95% CI: 0.9519–0.9674), which had high clinical value.^[27]^ Zhang et al used REO combined with mRMR and MRMD feature selection methods to develop the eHCC-pred model, in which the mRMR + SVM predictor showed good diagnostic ability with an AUC of 0.9384, an accuracy of 0.9834, and an F1 score of 0.9915.^[28]^ Lee et al constructed a clinical decision support system for HCC based on ML technology, and the C-index, AUC, and Brier scores of the internal and external datasets amounted to 0.8381 versus 0.7767, 91.89 versus 86.48, and 0.12 versus 0.14, respectively, which possessed a good value for clinical decision-making.^[29]^ In addition, Ravikulan et al explored the application of randomized survival forests (RSF) in predicting HCC recurrence risk. They compared it with the Cox proportional risk model (CPH), and the study demonstrated that RSF was superior to CPH in predicting HCC recurrence.^[30]^

In this context, the combination of gene transcriptome technology and ML helps to identify novel biomarkers and offers the possibility of building more accurate and personalized HCC prognostic models. In this study, an integrated approach based on the combination of 101 ML algorithms was used to systematically analyze the prognostic features of HCC based on key prognostic genes of HCC and combined with the analysis of immune infiltration, drug sensitivity, and tumor mutation load to enhance the robustness and accuracy of prognostic prediction. This study aims to identify key prognostic markers, optimize ML model combinations, and construct high-precision clinical prediction models to provide comprehensive prognostic analyses for HCC patients, which will guide clinical decision-making and individualized treatment.

## 2. Materials and methods

### 2.1. Data set sources

In this study, transcriptome data of 421 and 988 HCC tissue samples were obtained from The Cancer Genome Atlas (TCGA) and International Cancer Genome Consortium (ICGC) databases, respectively, and the corresponding clinical information of HCC patients was downloaded. In addition, gene expression data of 110 cases from the normal population were obtained from the GTEx database.

### 2.2. Limma differential expression analysis

This study used the “limma” package to perform differential analysis of the combined TCGA and GTEx data to screen for differentially expressed genes (DEGs). In the analysis, HCC samples were compared with normal liver tissues to screen for significantly DEGs, and volcano and heat maps of DEGs were plotted to visualize the differential analysis results. The criteria for differential expression were FDR-adjusted *P*-value <.05 and |log2 fold change| > 1 to ensure statistical significance and biological significance.

### 2.3. Univariate and multivariate analyses and combined multiple ML algorithms to screen key prognostic genes and clinical indicators

This study used univariate and multivariate Cox regression analyses to assess the relationship between gene expression, clinical indicators, and OS in HCC patients. Potential prognostic factors were first screened by univariate analysis, and variables with *P* values <.05 were considered statistically significant. Subsequently, these significant factors were included in multivariate Cox regression to identify independent prognostic genes and adjust for potential confounding variables. To improve the robustness of prognostic marker selection, this study combined 5 ML techniques, including random forest and LASSO regression, and the final screening results were intersected to ensure reliability and accuracy.

### 2.4. Optimal modeling algorithm and model evaluation for 101 combined ML screening

In this study, 101 combinatorial ML algorithms were innovatively applied to determine the optimal algorithmic model for liver cancer prognosis by utilizing the advantages of different models, reducing overfitting, and improving prediction performance through integrated learning. To ensure the robustness of the evaluation, TCGA data was used as the training set, ICGC data was used as the validation set, and 10-fold cross-validation was performed during training to assess the generalizability of the algorithms. The underlying algorithms used include Elastic Net (ENet), gradient boosting machine (GBM), supervised principal component analysis (SuperPC), stepwise Cox regression (StepCox), random survival forest (RSF), least absolute shrinkage and selection operator (Lasso), survival support vector machine (survival-SVM), ridge regression (Ridge), CoxBoost, partial least squares regression for Cox models (plsRcox).

Each algorithm is evaluated by several performance metrics, including the area under the receiver operating characteristic curve (AUC) and consistency index (C-index), which measure the model’s ability to discriminate between survival outcomes and time.^[31,32]^ By combining traditional statistical methods with advanced ML techniques, this study maximizes the potential of prognostic prediction models for liver cancer. This study further constructed the nomogram based on the best algorithms selected and their risk scores obtained from training. It verified the accuracy of the model and its potential application in clinical prediction by calibration curves, ROC curves, and DCA curves.

### 2.5. Feature importance analysis (based on SHAP technique)

In order to improve the interpretability of prognostic models and to assess the importance of prognostic markers, the shapley additive explanations (SHAP) method based on cooperative game theory was used in this study to compute the Shapley value of each feature to measure its contribution to model prediction.^[33]^ SHAP was applied to the filtered best combination algorithm model to assess the impact of each feature on risk prediction. The larger the absolute value of the SHAP value, the more significant the impact of the feature on prognostic prediction.

### 2.6. Analysis of key prognostic genes and immune cell infiltration in HCC patients

In this study, we analyzed the relationship between key genes and immune cell infiltration using immune cell deconvolution methods such as ESTIMATE, TIMER, ABIS, ConsensusTME, xCell, EPIC, quanTIseq, and CIBERSORT. The “ggplot2” package was used to calculate the gene expression and immune cell infiltration scores, which were visualized by bubble plots.

### 2.7. Drug sensitivity analysis of key prognostic genes

In this study, drug sensitivity analysis of key prognostic genes in HCC was performed using the OncoPredict method, which is based on gene expression profiling to predict the response of cancer cell lines to various anticancer drugs.^[34]^ By integrating gene expression data from HCC patients, we aim to mine potential therapeutic strategies against HCC progression, analyze drug sensitivity profiles to assess the efficacy of specific drugs, and provide personalized treatment plans for HCC patients.

### 2.8. HCC tumor mutation load analysis

This study analyzed the TMB of HCC and explored its relationship with key prognostic genes. The analysis used TCGA somatic mutation data (SNV) and gene expression profiles. TCGA mutation data were first downloaded, processed, and merged to calculate the TMB for each sample, that is, the number of mutations per megabase. Subsequently, the study performed a correlation analysis between TMB and the expression of specific genes to assess the relationship between mutation load and gene expression levels. The clinical relevance was also assessed by exploring the association of TMB with clinical characteristics such as age and survival status. The results were visualized by scatter plots, heat maps, and box plots to reveal further the impact of TMB on the prognosis and treatment response of HCC patients.

**Figure 1.**
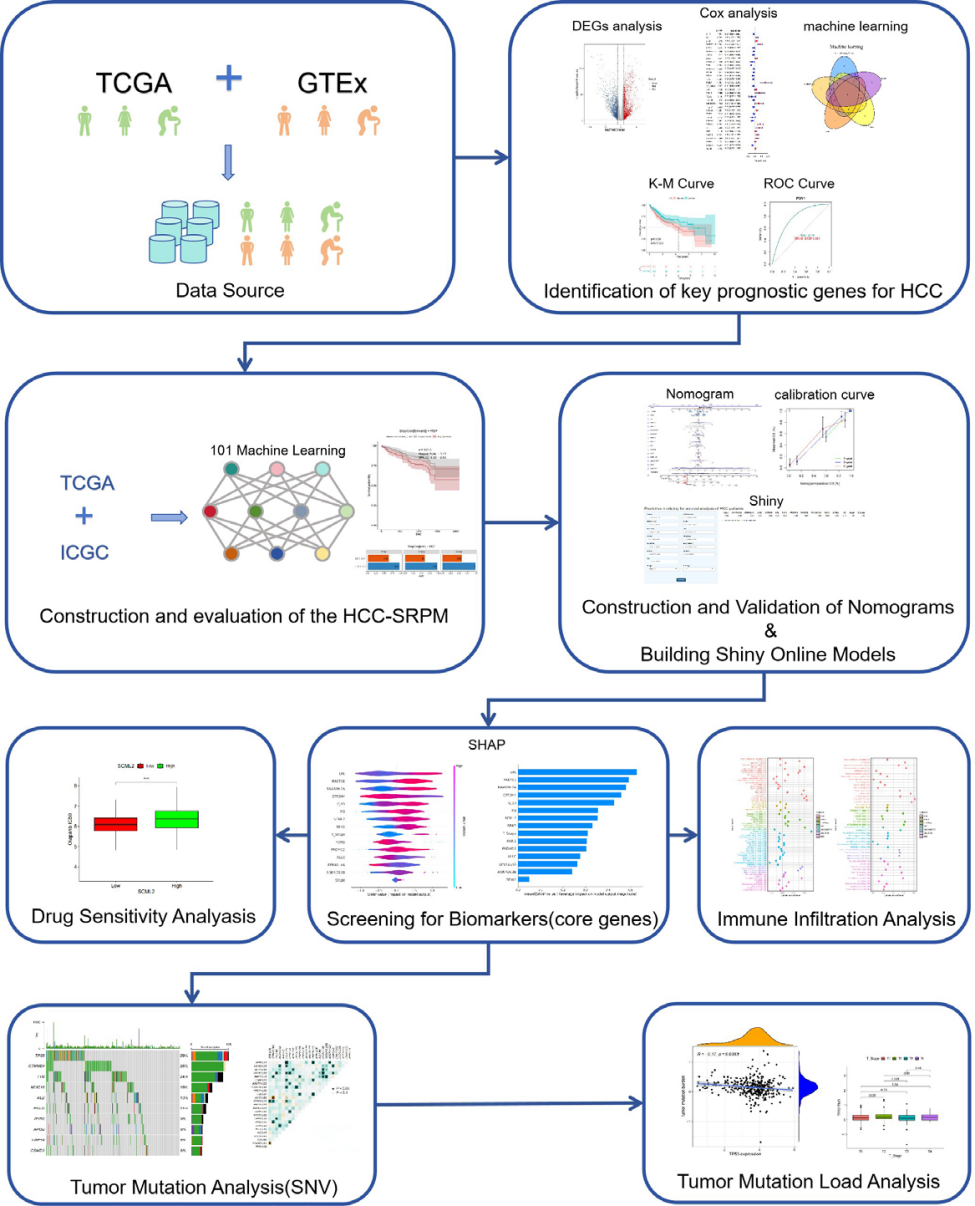
Flowchart of article techniques and methods.

Figure 1 details this study’s technical analysis process, demonstrating the complete process from data preprocessing to model interpretation.

## 3. Results

### 3.1. Screening of DEGs

In this study, we first preprocessed the HCC patient data obtained from the TCGA and GTEx databases. We performed PCA analysis on the data obtained from the 2 databases, respectively (as shown in Fig. S1C and E, Supplemental Digital Content, https://links.lww.com/MD/Q204). Subsequently, sample clustering and sample outlier segmentation were performed (TCGA outlier threshold = 415, 8 cases were excluded from the 421 samples; GTEx outlier threshold = 590, 6 cases were excluded from the 110 samples), as shown in Figure S1A and B (Supplemental Digital Content, https://links.lww.com/MD/Q204). In this study, PCA analysis was then performed for the preprocessed results, and the results are shown in Figure S1D and F (Supplemental Digital Content, https://links.lww.com/MD/Q204). Subsequently, limma differential expression analysis was performed on the TCGA data, and the results showed that a total of 4662 DEGs (2387 down-regulated expression and 2275 up-regulated expression) were detected, as shown in Figure 1A.

**Figure 2.**
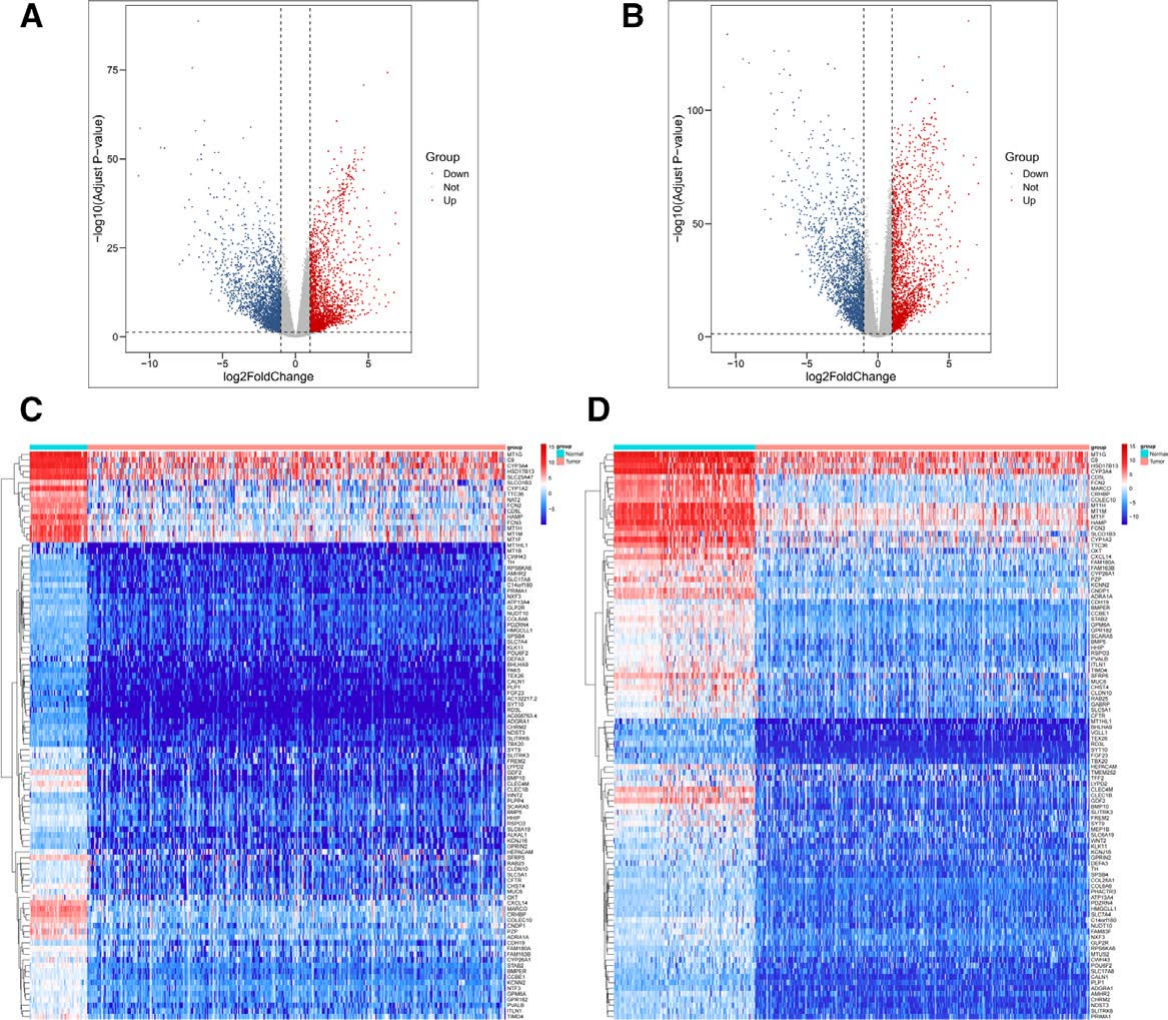
(A) Volcano plot of TCGA data differential genes. (B) Volcano plot of TCGA_GTEx differential genes. (C) Heat map of the top 100 DGEs of TCGA data. (D) Heatmap of the top 100 DGEs ranked by TCGA_GTEx. DEGs = differentially expressed genes, TCGA = the cancer genome atlas.

In order to expand the database and improve the accuracy and reliability of the analysis, the TCGA and GTEx data were combined in this study. The limma analysis was performed again after preprocessing (Fig. S2, Supplemental Digital Content, https://links.lww.com/MD/Q204), and 4312 DEGs (2272 down-regulated expression, 2040 up-regulated expression) were obtained, as shown in Figure 2B. In addition, the top 100 DEGs in TCGA and merged data are shown in Figure 2C and D, respectively. The results showed that the data preprocessing resulted in significant differences in gene expression between the normal and tumor groups, which provided a solid database for subsequent studies.

### 3.2. Screening for independent prognostic genes

**Figure 3.**
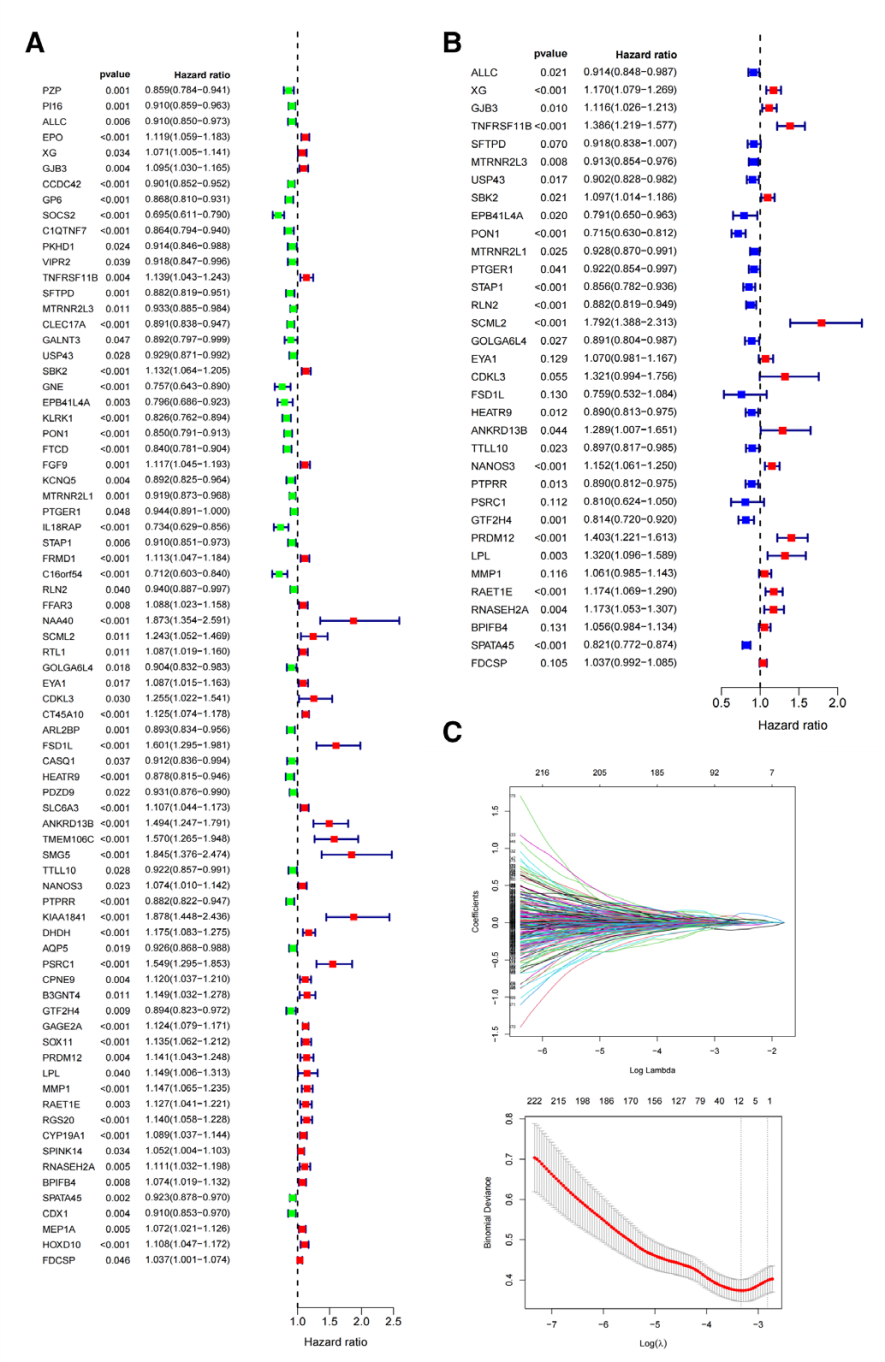
(A) Univariate analysis to screen for independent prognostic genes. (B) Multifactorial analysis to screen independent prognostic genes. (C) Lasso analysis to narrow down the independent prognostic genes.

To further identify and validate key independent prognostic genes, this study conducted a Cox proportional hazards analysis and then narrowed down the genes using lasso technology (see Fig. 3C and D). As shown in Figure 3A, for the resulting lasso output genes, this study conducted a univariate analysis, and then based on the results of the univariate analysis, this study conducted a multivariate analysis (see Fig. 3B), and the total number of genes with the independent prognostic value obtained from the multivariate analysis was 34, of which 18 genes had HR < 1 (ALLC, SFTPD, MTRNR2L3, USP43, EPB41L4A, PON1, MTRNR2L1, PTGER1, STAP1, RLN2, GOLGA6L4, FSD1L, HEATR9, TTLL10, PTPRR, PSRC1, GTF2H4, and SPATA45), which were independent risk genes; and 16 genes with HR > 1 (XG, GJB3, TNFRSF11B, SBK2, SCML2, EYA1, CDKL3, ANKRD13B, NANOS3, PRDM12, LPL, MMP1, RAET1E, RNASEH2A, BPIFB4, and FDCSP, respectively), were independent protective genes.

### 3.3. ML screening of feature genes

**Figure 4.**
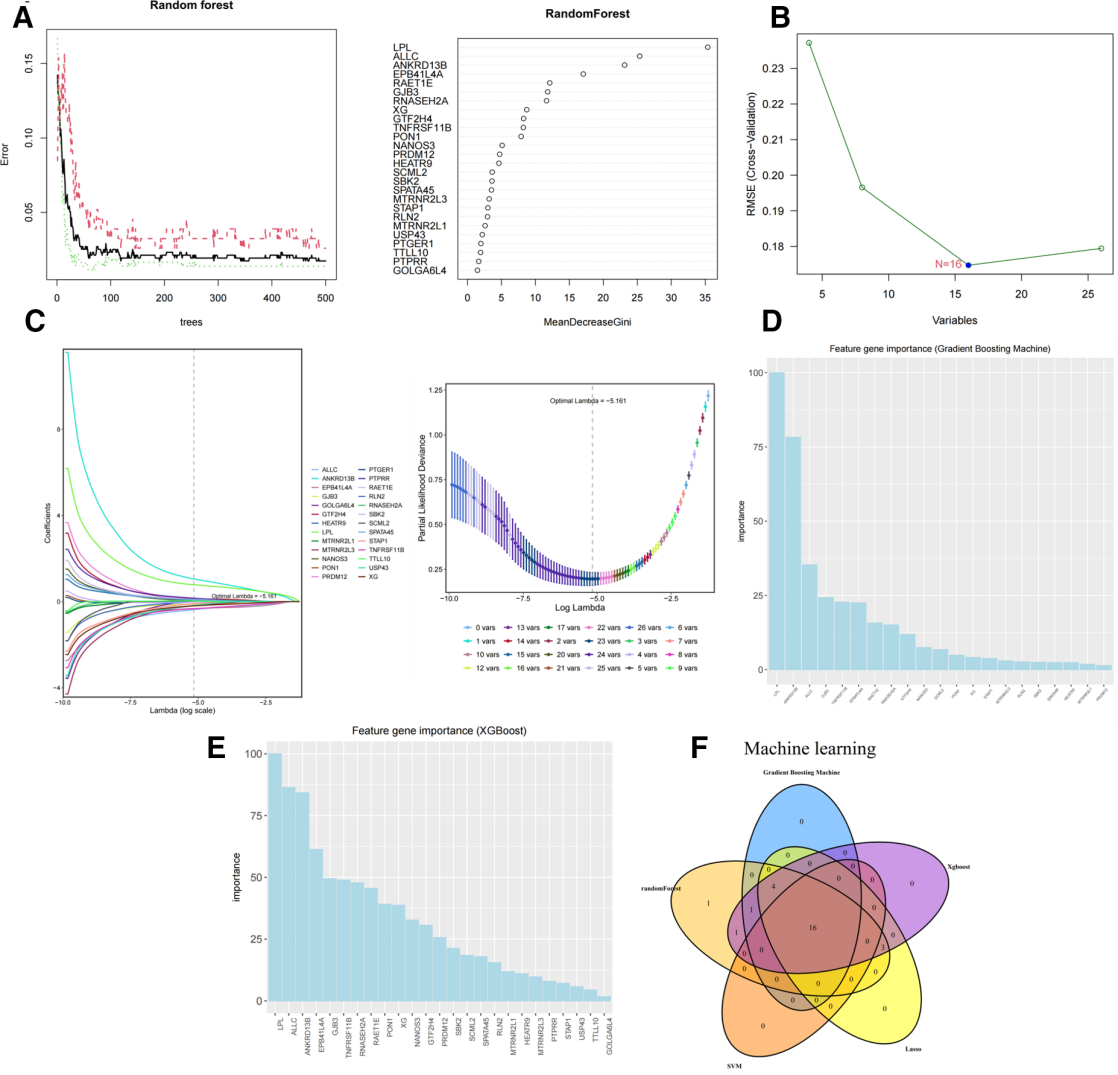
Multiple joint machine learning algorithms for screening prognostic genes. (A) Random forest. (B) SVM. (C) Lasso. (D) GBM. (E) XGBoost. (F) Venn diagram of 5 machine learning algorithms importance of the top sixteen genes.

In order to improve the accuracy of the screening results as well as the efficiency of the screening process, this study adopted several ML algorithms, such as random forest, SVM, lasso, GBM, and XGBoost, to screen the feature genes of HCC patients using a multi-algorithm joint approach, as shown in Figure 4A–E. In this study, based on the training results of the 5 ML models, the feature expression genes screened by them were taken as intersection, and finally, 16 feature genes were obtained, which were TNFRSF11B, NANOS3, SPATA45, LPL, ANKRD13B, ALLC, GJB3, EPB41L4A, RAET1E, RNASEH2A, GTF2H4, SCML2, PON1, XG, SBK2, PRDM12, as shown in Figure 4F.

### 3.4. Establishment of K–M survival curve model for disease gene expression analysis of characterized genes

**Figure 5.**
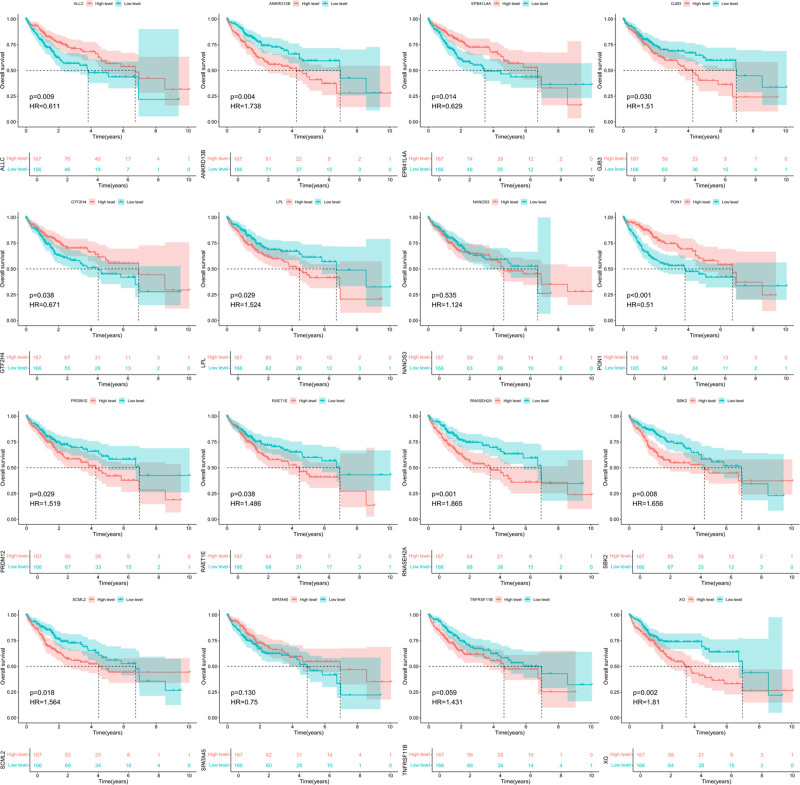
K–M survival curve analysis of key prognostic genes in HCC patients. HCC = hepatocellular carcinoma, K–M = Kaplan–Meier.

This study used the K–M (Kaplan–Meier) survival curve to analyze the correlation between the high-level and low-level expression of the 16 genes screened in HCC and to screen for characteristic genes. The analysis results are shown in Figure 5. Among the 16 key genes, the high and low-level expression of TNFRSF11B, NANOS3, and SPATA45 were not statistically significant in the prognosis of HCC patients (*P* > .05). While LPL, ANKRD13B, ALLC, GJB3, EPB41L4A, RAET1E, RNASEH2A, GTF2H4, SCML2, PON1, XG, SBK2, and PRDM12 possessed a significant differentiation and statistically significant correlation between high-level expression of the genes and low-level expression in prognosis of HCC patients (*P* < .05), these 13 genes were further screened as the key prognostic genes with HCC patients.

### 3.5. Study of diagnostic accuracy of prognostic key genes in HCC patients

**Figure 6.**
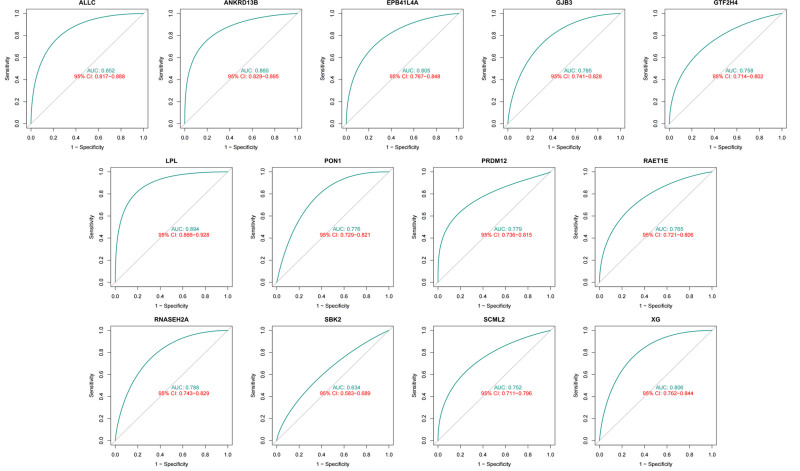
ROC curve analysis of key prognostic genes in HCC patients. HCC = hepatocellular carcinoma.

In this study, the key prognostic genes screened based on K–M curves were analyzed using the AUC values of ROC curves for 13 genes including LPL, ANKRD13B, ALLC, GJB3, EPB41L4A, RAET1E, RNASEH2A, GTF2H4, SCML2, PON1, XG, SBK2 and PRDM12, and their diagnostic value in the prognosis of HCC patients was evaluated. Figure 6 shows the above 13 key prognostic genes with an AUC value of > 0.75, indicated that the above-identified key HCC prognostic genes had significant diagnostic accuracy and predictive ability in the clinic and thus could be used as potential biomarkers for predicting the prognosis of HCC patients.

### 3.6. Establishment of baseline table to analyze patients’ clinicopathological information

This study conducted unifactorial and multifactorial analyses based on the Cox algorithm. The clinicopathological information of HCC patients was analyzed in detail, and the characteristics of the clinicopathological information of the patients in this study can be derived from Table 1. Among 224 HCC patients who met the inclusion criteria, the number of male patients with morbidity (67.9%, N = 152) exceeded the number of female patients (32.1%, N = 72); the incidence race was concentrated in Asians (63.8%, N = 143) and Caucasians (36.2%, N = 76); surviving patients with HCC had lower pathologic stage and T stage, concentrated in Stage I (56.2%, N = 86) and T1 (56.9%, N = 87), and deceased patients had higher pathologic stage and T stage, concentrated in Stage III (43.7%, N = 31) and T3 (36.6%, N = 26); the N-stage and M-stage of the HCC diseased population were highly concentrated in N0 and M0; the mean age of the deceased patients was lower than that of the surviving patients. Meanwhile, pathologic staging and T-staging possess statistical significance and will be used as variables for predictive model training.

**Table 1:** Baseline table of unifactorial and multifactorial analyses of clinicopathologic information in patients with HCC.

Characteristics	Variables	Alive	Dead	Univariate analysis		Multivariate analysis	
				HR (95% CI)	*P*-value	HR (95% CI)	*P*-value
Gender	Female	44 (28.8%)	28 (39.4%)	0.81 (0.45–1.1)	.150		
	Male	109 (71.2%)	43 (60.6%)				
Race	American Indian or Alaska Native	2 (1.3%)	0 (0%)	6.32 (0–12.63)	.391		
	Asian	102 (66.7%)	41 (57.7%)				
	Black or African American	2 (1.3%)	1 (1.4%)				
	White	47 (30.7%)	29 (40.8%)				
Stage	I	86 (56.2%)	23 (32.4%)	1.97 (0.82–3.1)	<.001	7.89 (0.04–15.7)	.540
	II	35 (22.9%)	14 (19.7%)				
	III	30 (19.6%)	31 (43.7%)				
	IV	2 (1.3%)	3 (4.2%)				
T	T1	87 (56.9%)	24 (33.8%)	1.85 (0.78–2.9)	<.001	43.52 (0.21–86.8)	.435
	T2	37 (24.2%)	14 (19.7%)				
	T3	26 (17%)	26 (36.6%)				
	T4	3 (2%)	7 (9.9%)				
N	N0	151 (98.7%)	69 (97.2%)	4.52 (0.51–8.5)	.801		
	N1	2 (1.3%)	2 (2.8%)				
M	M0	68 (95.8%)	220 (98.2%)	6.93 (1.24–12.2)	.182		
	M1	3 (4.2%)	4 (1.8%)				
Age	Mean ± SD	58.24 ± 13.76	56.93 ± 13.38	1.01 (0.99–1.03)	.321		

HCC = hepatocellular carcinoma.

### 3.7. Screening the best algorithm of shiny online prediction model based on 101 ML algorithms and building survival prognostic nomograms

**Figure 7.**
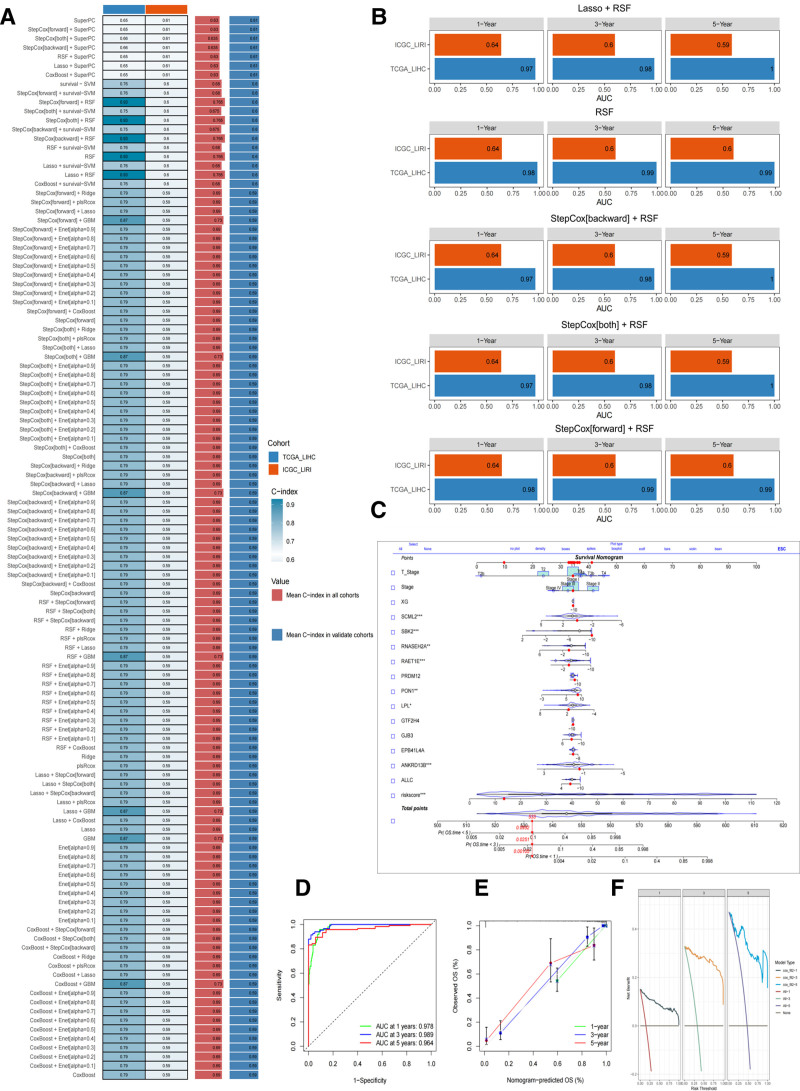
(A) C-index values of training results of 101 combined machine learning algorithms. (B) AUC values (training set + test set) at 1, 3, and 5 yr for models Lasso + RSF, RSF, StepCox[backward] + RSF, StepCox[both] + RSF, and StepCox[forward] + RSF. (C) Nomo prediction scales for HCC patients at 1, 3, and 5 yr. (D) ROC curves. (E) Calibration curves. (F) DCA curve. HCC = hepatocellular carcinoma, RSF = random survival forest, StepCox = stepwise Cox regression.

This study constructed a survival prediction model for HCC using ML techniques based on 13 key prognostic genes and their expression levels. Based on Enet and other algorithms to fit 101 combinatorial models and using the TCGA-LIHC cohort as the training set and the ICGC-LIRI cohort as the validation set, the C-index of each model was calculated (see Fig. 7A for details). The results screened 5 models with excellent performance: StepCox[forward] + RSF, StepCox[both] + RSF, StepCox[backward] + RSF, RSF, and Lasso + RSF, with an average C-index of 0.765 (right column of Fig. 7A). This result validated the robustness of the HCC scoring model constructed based on 13 genetic features.

AUC values were calculated for the 5 models to screen the best prediction models further. As shown in Figure 7B, among them, StepCox[forward] + RSF and RSF performed the best, with TCGA-LIHC cohort AUC (1 year: 0.98; 2 years: 0.99; 3 years: 0.99) and ICGC-LIRI cohort AUC (1 year: 0.64; 2 years: 0.60; 3 years: 0.60). Risk-stratified analyses (“high”; “low”) showed statistically significant K–M analyses for both StepCox[forward] + RSF and RSF models (TCGA-LIHC: *P* < .001; ICGC-LIRI: *P* = .013) (see Fig. S3, Supplemental Digital Content, https://links.lww.com/MD/Q204).

StepCox[forward] + RSF, although possessing greater model interpretability to clarify the effect of variables on survival time through *P*-values from Cox regression, the overall algorithmic architecture did not apply to predicting the risk of death. Therefore, RSF was finally selected to create the best HCC mortality risk prediction model, HCC-SRPM (survival risk prediction model for HCC patients).

As shown in Figure 7C, this study constructed nomograms for 1-, 3- and 5-year survival prediction based on RSF model-trained HCC patient risk scores and Cox multifactorial regression analysis, integrating 13 key genes, stage, and T-stage. The AUC of the model ROC curve was 0.978 (1 year), 0.989 (3 years), and 0.964 (5 years), which indicated its superior predictive performance and substantial clinical application value (see Fig. 7D). Meanwhile, this study was further validated using bootstrap (iteration = 1000), and the nomogram calibration curve showed that the actual values were in high agreement with the predicted values (Fig. 7E), which supports the reliability of the model in the prediction of short-to medium-term survival in HCC. In addition, the DCA curve assessment was shown in Figure 7F, and the clinical benefits of the model in 1-year, 3-year, and 5-year survival prediction were high, indicating its good robustness and generalization ability.

### 3.8. Shiny-based online clinical mortality risk prediction model for HCC

**Figure 8.**
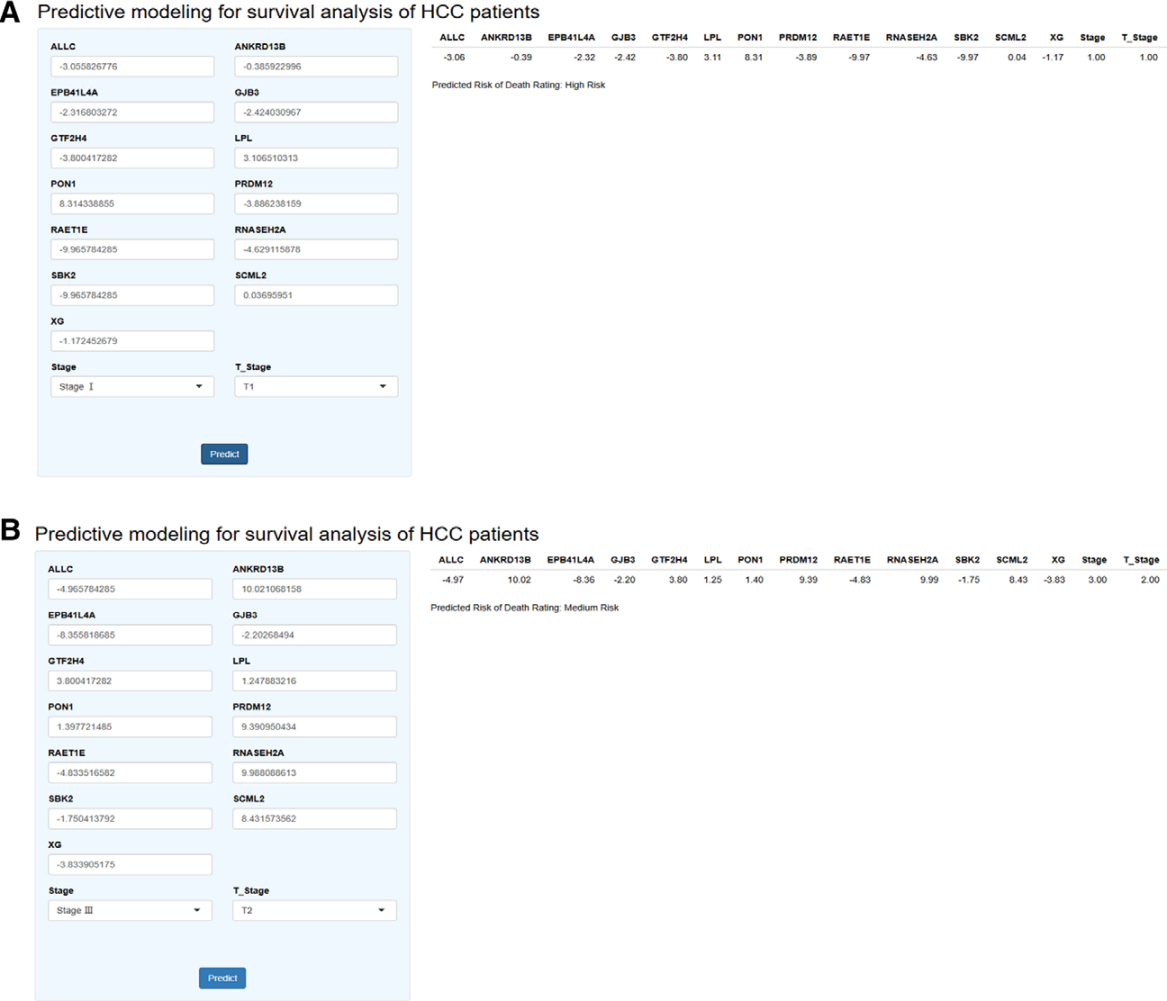
(A) Patients with HCC diagnosed at high risk of death. (B) HCC patients diagnosed at intermediate risk of death. HCC = hepatocellular carcinoma.

In this study, the Shiny application for survival risk prediction for HCC was developed based on Shiny to calculate patients’ mortality risk classes to maximize the translation of HCC-SRPM into a real-world clinical practice tool. As shown in Figure 8, A and B are patients with a moderate risk of death and patients with a high risk of death in real-world prediction. This online prediction model uses the best model RSF selected from 101 combinatorial ML and allows clinicians to calculate the individualized risk of death for HCC patients based on 15 key prognostic factors with significant biologic characteristics.

The application facilitates clinical research and daily practice. Clinicians can access the app via the link: predictive modeling for survival analysis of HCC patients (https://osteoporosispredictionmodel.shinyapps.io/HCC-improved-medic_predict/).

### 3.9. Importance analysis of features based on SHAP algorithm

**Figure 9.**
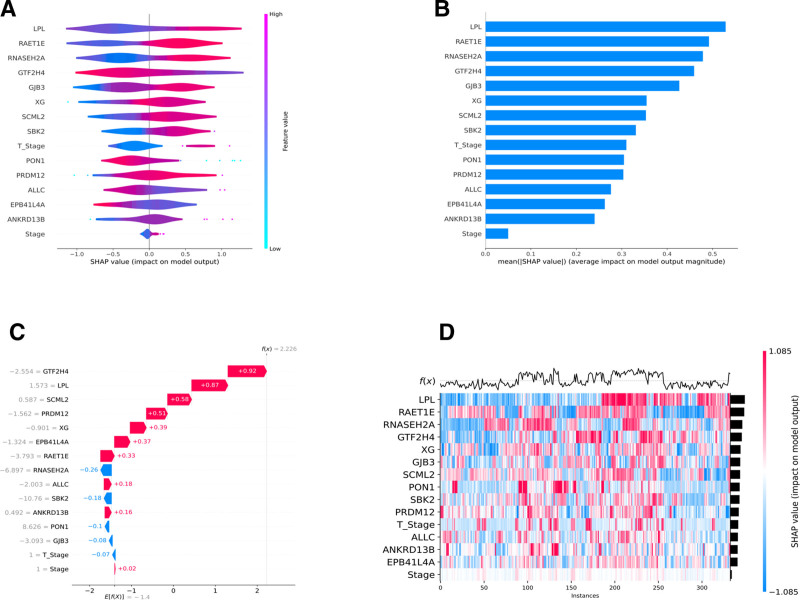
Visualization of key prognostic factors of RSF model based on SHAP analysis technique. (A) Summary plot. (B) Bar chart ranking features based on their average impact. (C) Waterfall plot. (D) Cross-instance SHAP value heatmap. RSF = random survival forest, SHAP = shapley additive explanations.

In this study, SHAP was applied to analyze the impact of specified features in the RSF model on the mortality of HCC patients. Figure 9A shows the importance ranking of the top 15 key predictors, including LPL, ANKRD13B, ALLC, GJB3, EPB41L4A, RAET1E, RNASEH2A, GTF2H4, SCML2, PON1, XG, SBK2, PRDM12, Stage, and T_Stage, and their mean values of significance are shown in Figure 9B.

As shown in Figure 9C, a waterfall plot was used in this study to visualize the contribution of each predictor to disease mortality, where GTF2H4, LPL, SCML2, PRDM12, XG, EPB41L4A, RAET1E, ALLC, ANKRD13B, and Stage had a positive contribution, and RNASEH2A, SBK2, PON1, GJB3 and T_Stage showed negative contribution (see Fig. 9D). Based on SHAP analysis, the top 4 key genes (LPL, RAET1E, RNASEH2A, GTF2H4, SCML2, and PRDM12) ranked in the 4 analyses were screened and summarized in this study as the core prognostic biomarkers and included in the subsequent prognostic analysis.

### 3.10. Analysis of immune infiltration of key prognostic genes in HCC patients

**Figure 10.**
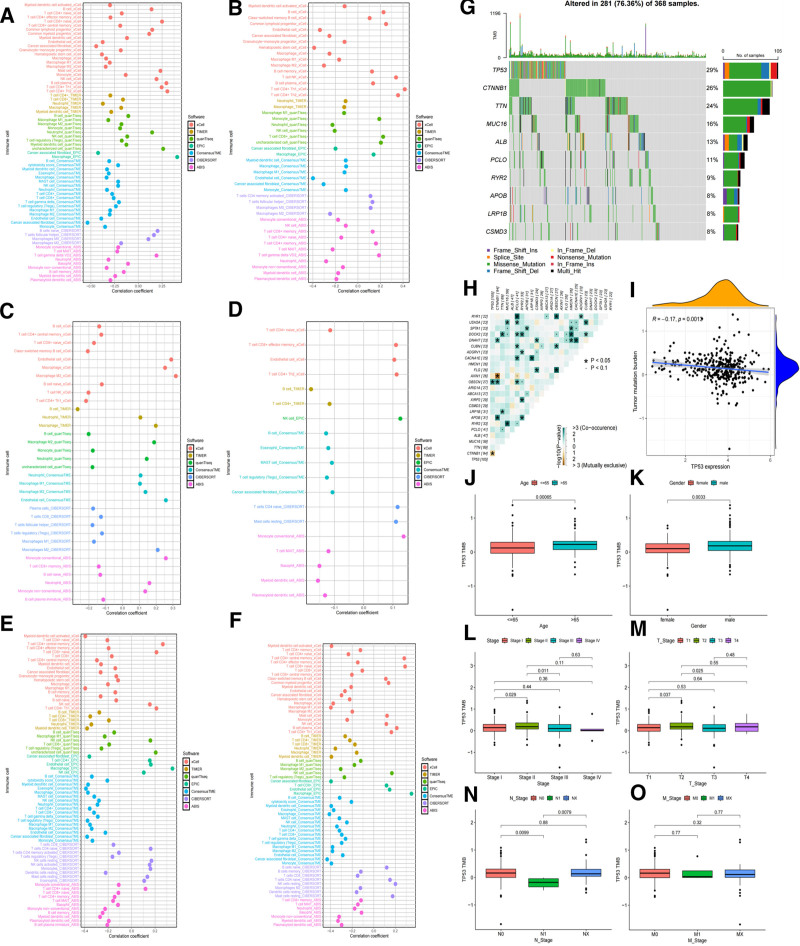
(A–F) Analysis of HCC prognostic key genes and immune cell infiltration in HCC patients. (G) Waterfall plot of HCC tumor mutation gene analysis. (H) Heatmap of HCC tumor interactions. (I) HCC tumor mutation load analysis (TP53). (J) Correlation analysis of TP53 expression with age. (K) Correlation analysis of TP53 expression with gender. (L) Correlation analysis of TP53 expression with cancer stage. (M) Correlation analysis of TP53 expression with T stage. (N) Correlation analysis of TP53 expression with lymphatic metastasis. (O) Correlation analysis of TP53 expression with distant metastasis. HCC = hepatocellular carcinoma.

This study used a multi-algorithm joint analysis to explore the relationship between HCC prognostic key genes and immune cell infiltration. As shown in Figure 10, LPL, RAET1E, RNASEH2A, GTF2H4, SCML2, and PRDM12 all play important roles in immune regulation. As shown in Figure 10A–F, SCML2 was positively correlated with macrophage (Mφ [1]) infiltration (0.375 ≤ *R* ≤ 0.500) and negatively correlated with tumor-associated fibroblasts (CAFs) infiltration (−0.625 ≤ *r* ≤ −0.500); RNASEH2A was positively correlated with helper T cell 1 (TH1) infiltration (0.400 ≤ *R* ≤ 0.450) and negatively correlated with endothelial cell (EC) infiltration (−0.450 ≤ *r* ≤ −0.350); LPL was positively correlated with M2 macrophage (M2) infiltration (0.300 ≤ *R* ≤ 0.350) and negatively correlated with B cell (B cell) infiltration (−0.300 ≤ *r* ≤ −0.250); RAET1E was positively correlated with conventional monocyte (cMo) infiltration (0.100 ≤ *R* ≤ 0.150) and negatively correlated with B cell (B cell) infiltration (−0.200 ≤ *r* ≤ −0.150); PRDM12 was positively correlated with macrophage (Mφ[1]) infiltration (0.250 ≤ *R* ≤ 0.300), and negatively correlated with M1-type macrophage (M1) infiltration (−0.450 ≤ *r* ≤ −0.300); GTF2H4 was positively correlated with macrophage (Mφ[1]) infiltration (0.300 ≤ *R* ≤ 0.400) and negatively correlated with CAFs infiltration (−0.470 ≤ *r* ≤ −0.400).

### 3.11. HCC tumor mutation gene load analysis and clinical correlation analysis

In this study, we mapped the tumor mutation profiles of HCC samples (see Fig. 10G). We analyzed genomic features such as somatic mutations and copy number variations based on the TCGA-LIHC dataset. The results showed a high frequency of TP53 (29%), CTNNB1 (26%), TTN (24%), and MUC16 (16%) mutations.

The mutated genes were further analyzed for mutual exclusivity and covariance to explore the interactions between TP53 and other gene mutations. Figure 10H shows that there was a covariance between TP53 and OBSCN (*P* < .05, −log10(*P*-value) > 2), while there was a mutual exclusivity between TP53 and CTNNB1 (*P* < .05, −log10(*P*-value) > 1.5). A weak negative correlation was observed between TP53 expression levels and tumor mutational load (TMB) (*R* = −0.17, *P* = .0013, see Fig. 10I), suggesting that TP53 may play a role in the immune microenvironment of HCC.

In this study, we further investigated the relationship between TP53 expression levels and clinicopathologic features of HCC based on TCGA data. Figure 10J–O shows that TP53 expression levels were significantly higher in patients aged > 65 years than in those aged ≤ 65 years (*P* = .00065) and higher in men than in women (*P* = .0033); in addition, TP53 expression levels were correlated with HCC histologic stages, with significant differences between Stage I and Stage II (*P* = .029) and Stage II and Stage III (*P* = .011), and Stage II expression levels were higher than those of Stage I and Stage III, and with the progression of staging, TP53 expression showed a decreasing trend; TP53 expression level was also correlated with T stage, and there were significant differences between T1 and T2 (*P* = .037) and T2 and T3 (*P* = .025), and the expression level of T2 was higher than that of T1 and T3; in addition, TP53 expression was significantly higher in patients without lymph node metastasis (N0) than in N1 (*P* = .0099); however, TP53 was not significantly associated with distant metastasis in HCC patients.

### 3.12. Drug sensitivity analysis of key prognostic genes in HCC patients

In this study, drug sensitivity analysis of 6 key prognostic genes in HCC was performed to assess specific drugs’ potential therapeutic effects and provide individualized treatment strategies for HCC patients. As shown in Tables S1–S4 (Supplemental Digital Content, https://links.lww.com/MD/Q205), the 4 genes differed in some drugs, but the number of differences and sensitivities for some existing drugs were insignificant. As shown in Table 2, SCML2 gene expression levels significantly affected the sensitivity of multiple chemotherapy and targeted therapy drugs. The low-expression group was more sensitive to most of the tested drugs. In contrast, the high-expression group was more sensitive to Navitoclax, suggesting that SCML2 could serve as a potential biomarker for therapeutic decision-making, guiding drug selection for patients with different expressions. As shown in Table 3, the low-expression group of the PRDM12 gene was more sensitive to Camptothecin, Cisplatin, Cytarabine, Docetaxel, Navitoclax, Olaparib, and Vinblastine. In contrast, the high-expression group was more sensitive to Nilotinib. This result suggests that PRDM12 gene expression level is clinically important in developing HCC prognosis and treatment strategy.

**Table 2:** Drug sensitivity analysis form (SCML2).

Drug	IC50-value (low group)	IC50-value (high group)	*P*-value
Camptothecin	0.124	0.184	<.001
Cisplatin	4.526	4.867	<.001
Ytarabine	2.465	2.997	<.001
Docetaxel	0.015	0.018	<.001
Gefitinib	4.625	4.814	<.001
Navitoclax	3.023	2.870	<.050
Nilotinib	5.215	5.228	1.000
Olaparib	6.086	6.337	<.001
Vinblastine	0.035	0.043	<.001
Vorinostat	2.379	2.392	1.000

**Table 3:** Drug sensitivity analysis form (PRDM12).

Drug	IC50-value (low group)	IC50-value (high group)	*P*-value
Camptothecin	0.135	0.173	<.001
Cisplatin	4.421	4.972	<.001
Cytarabine	2.572	2.890	<.001
Docetaxel	0.016	0.018	<.050
Gefitinib	4.699	4.740	1.000
Navitoclax	2.887	3.007	<.050
Nilotinib	5.357	5.084	<.001
Olaparib	6.029	6.396	<.001
Vinblastine	0.036	0.042	<.050
Vorinostat	2.407	2.363	1.000

## 4. Discussion

This study used PCA dimensionality reduction, clustering, and outlier analysis to improve TCGA and GTEx data quality. Subsequently, limma difference analysis was used to identify DEGs in the data, and candidate genes were screened by Lasso regression, which was combined with Cox one-way and multifactorial regression analyses to identify 34 independent prognostic genes. On this basis, 5 ML algorithms were used to screen and identify 16 core genes closely associated with HCC occurrence and death further. After K–M survival analysis and ROC curve validation, 13 key prognostic genes were finally identified: LPL, ANKRD13B, ALLC, GJB3, EPB41L4A, RAET1E, RNASEH2A, GTF2H4, SCML2, PON1, XG, SBK2 and PRDM12.

In this study, we further investigated the impact of clinicopathologic information on HCC prognosis and screened the best prognostic models based on 101 combinatorial ML algorithms. Then, the nomograms for 1-year, 3-year, and 5-year survival prediction of HCC patients were constructed based on the risk scores derived from the model training, in which the AUC values of 1-year, 3-year and 5-year were 0.978, 0.989 and 0.964, respectively. Meanwhile, the DCA and calibration curves were well-behaved, demonstrating that the model developed in the present study possessed excellent clinical prediction ability and value for use.

Presently, the prediction of mortality risk and prognosis assessment of liver cancer is still the focus of clinical diagnosis and treatment. However, the robustness, broad applicability, and prediction accuracy of the existing models are still insufficient, and some models’ application is controversial.^[35]^ With the development of sequencing technology, the combined application of ML and biomarkers has shown promising results in HCC prediction.^[36]^ Based on this, this study included TCGA database patients (survival status and survival time) as the training set and ICGC database patients as the test set and used 13 key prognostic genes as the feature variables to construct 101 combined prediction models based on 10 ML algorithms, and evaluated the model performance by AUC, C-index, and other indicators.

The comprehensive evaluation showed that the RSF model had the best prediction effect. After incorporating the 13 key genes, its training set AUC (1 year, 3 years, 5 years) was 0.98, 0.99, 0.99, and the test set AUC was 0.64, 0.60, and 0.60, respectively. The model’s C-index, (OOB) CRPS, (OOB) stand. CRPS and (OOB) Requested performance errors were 0.765, 575.65, 0.1842, and 0.2849, respectively, indicating that the model is robust, has high prediction accuracy and very low error, and has high clinical application value. To ensure the robustness of the model, this study developed an online survival risk prediction tool based on the R package “Shiny,” which can be accessed via the link (https://osteoporosispredictionmodel.shinyapps.io/HCC-improved-medic_predict/). It provides support for individualized diagnosis and treatment of HCC.

In this study, we further used SHAP technology to parse the effects of key features in the RSF model on the risk of death in HCC patients, to validate the robustness of the modeling algorithm, and to screen 6 core prognostic genes (LPL, RAET1E, RNASEH2A, GTF2H4, SCML2, and PRDM12) as the basis of the subsequent study. All 6 genes are core prognostic markers for HCC, consistent with previous studies.^[37–42]^ To further explore the impact of the 6 core prognostic genes on the prognosis of HCC, the present study combined multiple algorithms for immune infiltration analysis and found that these genes were closely associated with the infiltration levels of macrophages, tumor-associated fibroblasts, T cells, ECs, B cells, and conventional monocytes. It was shown that tumor-associated fibroblasts and tumor-associated macrophages can interact in the HCC TME through the bone-bridging protein pathway and extensively infiltrate HCC tissues.^[43]^ In addition, a study by Garnelo et al indicated that the interaction between tumor-infiltrating T cells and B cells enhances local immune activation and improves the prognosis of HCC patients.^[44]^ It has been shown that tumor-derived endothelial cells (TEC) have enhanced angiogenic activity, mediated chemotherapy, and anti-angiogenic inhibitor resistance, providing a potential target for anti-angiogenic therapy in HCC.^[45]^ In addition, Tianyu Li et al found that the levels of myeloid-derived suppressor cells (MDSCs) and neutrophil-like MDSCs (PMN-MDSCs) were significantly higher in HCC patients than in patients with chronic hepatitis B (CHB) and healthy controls, and were significantly correlated with indirect bilirubin, prealbumin, systemic inflammatory response index, and monocyte/lymphocyte ratio,^[46]^ providing theoretical support for the immune infiltration analysis in this study.

In addition, this study evaluated the sensitivity of 6 core prognostic genes to ten chemotherapeutic and targeted drugs and found that the expression levels of SCML2 and PRDM12 significantly impacted the efficacy of several antitumor drugs. The SCML2 low-expression group significantly correlated to most drugs tested, while the high-expression group was more sensitive to Navitoclax. Patients with low PRDM12 expression were more sensitive to Camptothecin, Cisplatin, Cytarabine, Docetaxel, Navitoclax, Olaparib, and Vinblastine, while patients with high expression were more sensitive to NIL. These results suggest that gene expression of SCML2 and PRDM12 can provide an important reference for the individualized treatment of HCC patients, consistent with Peng and Liu et al.^[42,47]^

Studies have shown that the molecular pathogenesis of HCC involves a variety of genetic and epigenetic changes; for example, chronic infection of hepatitis B virus and hepatitis C virus, as well as oxygen radical diseases, including hemochromatosis, can induce the production of reactive oxygen/nitrogen species, which not only lead to DNA damage, but also may trigger the mutation of oncogenes, such as TP53, and thus play a key role in the molecular pathogenesis of HCC.^[48]^ In this study, based on the TCGA-LIHC dataset, we systematically analyzed genomic features such as somatic mutations and copy number variation in HCC to explore the mutations of different genes in HCC. The results showed that the somatic mutation frequencies of TP53, CTNNB1, TTN, and MUC16 genes were high, which were 29%, 26%, 24%, and 16%, respectively. Further mutual exclusivity and covariance analyses of tumor-mutated genes were performed, and it was found that TP53 showed a symbiotic relationship with OBSCN and exhibited mutual exclusivity with CTNNB1.

In addition, this study investigated the relationship between TP53 gene expression level and tumor mutation load (TMB), and the results showed that TP53 expression was weakly negatively correlated with TMB (*R* = −0.17, *P* = .0013). Woo HG et al demonstrated that the level of TP53 expression was correlated with clinical characteristics such as tumor stage of HCC patients and had a significant impact on the survival rate of patients.^[49–54]^ Based on this, this study further analyzed the expression characteristics of TP53 in HCC patients and its association with clinicopathological factors. The results showed that the expression level of TP53 was significantly higher in patients ≥ 65 years old than in patients < 65 years old (*P* = .00065). Meanwhile, there was also a significant difference in the gene between genders, with higher expression levels in men than in women (*P* = .0033). In terms of histologic staging, there were significant differences in TP53 expression levels between Stage I and II (*P* = .029) and between stage II and III (*P* = .011), with the expression levels in stage II higher than those in stage I and III, and showing a decreasing trend with the progression of staging. In addition, TP53 expression was significantly correlated with T staging, with higher expression levels in T2 than in T1 (*P* = .037) and T3 (*P* = .025). In contrast, the differences in other T staging periods were not statistically significant. Meanwhile, there was a significant difference in the expression of this gene between the N0 and N1 stages (*P* = .0099), in which the expression level was higher in the N0 stage than in the N1 stage, suggesting that TP53 may be associated with lymph node metastasis. However, no significant association between TP53 expression and distant metastasis was found in this study.

This study screened 13 key prognostic genes and constructed HCC-SRPM based on 101 machine-learning algorithms. Finally, the optimal model RSF was screened, and an online clinical prediction tool was developed. In this study, SHAP was further used to parse the contribution of model features. Six HCC core prognostic genes (LPL, RAET1E, RNASEH2A, GTF2H4, SCML2, and PRDM12) were ultimately identified and subjected to immune infiltration analysis, which showed that these genes were significantly correlated with the tumor microenvironmental cells, such as T-cells, macrophages, and B-cells. In addition, SCML2 and PRDM12 showed high sensitivity to various antitumor drugs, demonstrating their potential value in HCC-targeted therapy. This study further reveals that multiple factors, including clinicopathologic features such as lymph node metastasis, Stage, T stage, age, and gender, influence the prognosis of HCC. However, this study still has some limitations. First, this study was based on data from public databases, and future multicenter clinical studies must be combined to incorporate more detailed patient information to optimize the model and improve clinical prediction performance. Second, the model constructed in this study is still in the theoretical stage and needs to be further validated by large-scale clinical trials to improve its generalization ability and clinical applicability.

## 5. Conclusion

In this study, LPL, RAET1E, RNASEH2A, GTF2H4, SCML2, and PRDM12 were screened as potential diagnostic and prognostic markers of HCC using various algorithmic techniques, of which SCML2 and PRDM12 were significantly correlated with multiple drugs in drug sensitivity analysis. TP53 mutations were associated with age, gender, tumor histological stage, T stage, and lymph node metastasis. In addition, we used ten ML algorithms, including ENet, GBM, SuperPC, StepCox, RSF, Lasso, survival-SVM, Ridge, CoxBoost, and plsRcox, to construct a total of 101 models for predicting the risk of death of patients with HCC, in order to evaluate the value of the application of ML in clinical prediction. The study shows that the models perform well in key prognostic gene screening and death risk prediction, among which the RSF model performs best in HCC diagnosis, with optimal classification effect and robustness, and exhibits high prediction performance. Meanwhile, a Shiny-based online prediction tool was developed to assist clinicians in optimizing treatment plans, reducing medical costs, and improving patient prognosis.

## Author contributions

**Conceptualization:** Jiaming Wang, TongPing Shen.

**Project administration:** Shihao Wang.

## Supplementary Material



## References

[1] Sung H Ferlay J Siegel RL. Global cancer statistics 2020: GLOBOCAN estimates of incidence and mortality worldwide for 36 cancers in 185 countries. CA Cancer J Clin. 2021;71:209–49. 33538338 10.3322/caac.21660

[2] GBD 2021 Nervous System Disorders Collaborators. Global, regional, and national burden of disorders affecting the nervous system, 1990–2021: a systematic analysis for the global burden of disease study 2021. Lancet Neurol. 2024;23:344–81. 38493795 10.1016/S1474-4422(24)00038-3 PMC10949203

[3] Kalantari L Ghotbabadi ZR Gholipour A. A state-of-the-art review on the NRF2 in Hepatitis virus-associated liver cancer. Cell Commun Signal. 2023;21:318. 37946175 10.1186/s12964-023-01351-6 PMC10633941

[4] Jacob R Prince DS Kench C Liu K. Alcohol and its associated liver carcinogenesis. J Gastroenterol Hepatol. 2023;38:1211–7. 37263779 10.1111/jgh.16248

[5] Sealock T Sharma S. Smoking Cessation (Archived). In: StatPearls. Treasure Island (FL): StatPearls Publishing; June 25, 2024. 29494049

[6] Pugliese N Alfarone L Arcari I. Clinical features and management issues of NAFLD-related HCC: what we know so far. Expert Rev Gastroenterol Hepatol. 2023;17:31–43. 36576057 10.1080/17474124.2023.2162503

[7] Petrick JL McGlynn KA. The changing epidemiology of primary liver cancer. Curr Epidemiol Rep. 2019;6:104–11. 31259140 10.1007/s40471-019-00188-3 PMC6599615

[8] Peña-Asensio J Calvo H Torralba M Miquel J Sanz-de-Villalobos E Larrubia JR. Anti-PD-1/PD-L1 based combination immunotherapy to boost antigen-specific CD8+ T cell response in hepatocellular carcinoma. Cancers (Basel). 2021;13:1922. 33923463 10.3390/cancers13081922 PMC8073815

[9] Lv W Li T Wang S. The application of the CRISPR/Cas9 system in the treatment of hepatitis B liver cancer. Technol Cancer Res Treat. 2021;20:15330338211045206. 34605326 10.1177/15330338211045206 PMC8493308

[10] Chang MH. Prevention of hepatitis B virus infection and liver cancer. Recent Results Cancer Res. 2021;217:71–90. 33200362 10.1007/978-3-030-57362-1_4

[11] Ji D Chen Y Bi J. Entecavir plus Biejia–Ruangan compound reduces the risk of hepatocellular carcinoma in Chinese patients with chronic hepatitis B. J Hepatol. 2022;77:1515–24. 35985545 10.1016/j.jhep.2022.07.018

[12] Hsu YC Huang DQ Nguyen MH. Global burden of hepatitis B virus: current status, missed opportunities and a call for action. Nat Rev Gastroenterol Hepatol. 2023;20:524–37. 37024566 10.1038/s41575-023-00760-9

[13] Wang Y Deng B. Hepatocellular carcinoma: molecular mechanism, targeted therapy, and biomarkers. Cancer Metastasis Rev. 2023;42:629–52. 36729264 10.1007/s10555-023-10084-4

[14] Woller N Engelskircher SA Wirth T Wedemeyer H. Prospects and challenges for T cell-based therapies of HCC. Cells. 2021;10:1651. 34209393 10.3390/cells10071651 PMC8304292

[15] Huang J Wu Q Geller DA Yan Y. Macrophage metabolism, phenotype, function, and therapy in hepatocellular carcinoma (HCC). J Transl Med. 2023;21:815. 37968714 10.1186/s12967-023-04716-0 PMC10652641

[16] Jeng LB Liao LY Shih FY Teng CF. Dendritic-cell-vaccine-based immunotherapy for hepatocellular carcinoma: clinical trials and recent preclinical studies. Cancers (Basel). 2022;14:4380. 36139542 10.3390/cancers14184380 PMC9497058

[17] Dai K Wu Y She S Zhang Q. Advancement of chimeric antigen receptor-natural killer cells targeting hepatocellular carcinoma. World J Gastrointest Oncol. 2021;13:2029–37. 35070039 10.4251/wjgo.v13.i12.2029 PMC8713322

[18] Khan MN Mao B Hu J. Tumor-associated macrophages and CD8+ T cells: dual players in the pathogenesis of HBV-related HCC. Front Immunol. 2024;15:1472430. 39450177 10.3389/fimmu.2024.1472430 PMC11499146

[19] Qi YQ Xiong F Chen YJ. The correlation between tumor-associated macrophages and the prognosis of east Asian hepatocellular carcinoma patients: a systematic review and meta-analysis. Pathol Res Pract. 2023;252:154919. 37939428 10.1016/j.prp.2023.154919

[20] Lip GY Nieuwlaat R Pisters R Lane DA Crijns HJ. Refining clinical risk stratification for predicting stroke and thromboembolism in atrial fibrillation using a novel risk factor-based approach: the Euro Heart Survey on atrial fibrillation. Chest. 2010;137:263–72. 19762550 10.1378/chest.09-1584

[21] O’Mahony C Jichi F Pavlou M; Hypertrophic Cardiomyopathy Outcomes Investigators. A novel clinical risk prediction model for sudden cardiac death in hypertrophic cardiomyopathy (HCM risk-SCD). Eur Heart J. 2014;35:2010–20. 24126876 10.1093/eurheartj/eht439

[22] Watson DS Krutzinna J Bruce IN. Clinical applications of machine learning algorithms: beyond the black box. BMJ. 2019;364:l886. 30862612 10.1136/bmj.l886

[23] Guan WJ Jiang M Gao YH. Unsupervised learning technique identifies bronchiectasis phenotypes with distinct clinical characteristics. Int J Tuberc Lung Dis. 2016;20:402–10. 27046724 10.5588/ijtld.15.0500

[24] Howard R Rattray M Prosperi M Custovic A. Distinguishing asthma phenotypes using machine learning approaches. Curr Allergy Asthma Rep. 2015;15:38. 26143394 10.1007/s11882-015-0542-0 PMC4586004

[25] Handelman GS Kok HK Chandra RV Razavi AH Lee MJ Asadi H. eDoctor: machine learning and the future of medicine. J Intern Med. 2018;284:603–19. 30102808 10.1111/joim.12822

[26] Bogard N Linder J Rosenberg AB Seelig G. A deep neural network for predicting and engineering alternative polyadenylation. Cell. 2019;178:91–106.e23. 31178116 10.1016/j.cell.2019.04.046 PMC6599575

[27] Zhang ZM Tan JX Wang F Dao FY Zhang ZY Lin H. Early diagnosis of hepatocellular carcinoma using machine learning method. Front Bioeng Biotechnol. 2020;8:254. 32292778 10.3389/fbioe.2020.00254 PMC7122481

[28] Zhang ZM Huang Y Liu G. Development of machine learning-based predictors for early diagnosis of hepatocellular carcinoma. Sci Rep. 2024;14:5274. 38438393 10.1038/s41598-024-51265-7 PMC10912761

[29] Lee KH Choi GH Yun J. Machine learning-based clinical decision support system for treatment recommendation and overall survival prediction of hepatocellular carcinoma: a multi-center study. NPJ Digit Med. 2024;7:2. 38182886 10.1038/s41746-023-00976-8 PMC10770025

[30] Ravikulan A Rostami K. Leveraging machine learning for early recurrence prediction in hepatocellular carcinoma: a step towards precision medicine. World J Gastroenterol. 2024;30:424–8. 38414588 10.3748/wjg.v30.i5.424 PMC10895597

[31] Streiner DL Cairney J. What’s under the ROC? An introduction to receiver operating characteristics curves. Can J Psychiatry. 2007;52:121–8. 17375868 10.1177/070674370705200210

[32] Antolini L Boracchi P Biganzoli E. A time‐dependent discrimination index for survival data. Stat Med. 2005;24:3927–44. 16320281 10.1002/sim.2427

[33] Van den Broeck G Lykov A Schleich M Suciu D. On the tractability of SHAP explanations. J Artif Intell Res. 2022;74:851–86.

[34] Maeser D Gruener RF Huang RS. oncoPredict: an R package for predicting in vivo or cancer patient drug response and biomarkers from cell line screening data. Brief Bioinform. 2021;22:bbab260. 34260682 10.1093/bib/bbab260 PMC8574972

[35] Cigliano A Liao W Deiana GA Rizzo D Chen X Calvisi DF. Preclinical models of hepatocellular carcinoma: current utility, limitations, and challenges. Biomedicines. 2024;12:1624. 39062197 10.3390/biomedicines12071624 PMC11274649

[36] Fa B Luo C Tang Z Yan Y Zhang Y Yu Z. Pathway-based biomarker identification with crosstalk analysis for robust prognosis prediction in hepatocellular carcinoma. EBioMedicine. 2019;44:250–60. 31101593 10.1016/j.ebiom.2019.05.010 PMC6606892

[37] Wu Z Ma H Wang L. Tumor suppressor ZHX2 inhibits NAFLD-HCC progression via blocking LPL-mediated lipid uptake. Cell Death Differ. 2020;27:1693–708. 31740790 10.1038/s41418-019-0453-z PMC7206072

[38] Long M Zhou Z Wei X. A novel risk score based on immune-related genes for hepatocellular carcinoma as a reliable prognostic biomarker and correlated with immune infiltration. Front Immunol. 2022;13:1023349. 36353638 10.3389/fimmu.2022.1023349 PMC9637590

[39] Hao Y Zou R Tao J Jiang M Li D. SP1/RNASEH2A accelerates the development of hepatocellular carcinoma by regulating EMT. Heliyon. 2023;9:e18127. 37520960 10.1016/j.heliyon.2023.e18127 PMC10374915

[40] Wang F Anderson PW Salem N Kuang Y Tennant BC Lee Z. Gene expression studies of hepatitis virus-induced woodchuck hepatocellular carcinoma in correlation with human results. Int J Oncol. 2007;30:33–44. 17143510

[41] Du L Wang L Yang H. Sex comb on midleg like-2 accelerates hepatocellular carcinoma cell proliferation and metastasis by activating Wnt/β-catenin/EMT signaling. Yonsei Med J. 2021;62:1073–82. 34816637 10.3349/ymj.2021.62.12.1073 PMC8612862

[42] Liu Y Ji H Wu LH. Stratifying hepatocellular carcinoma based on immunophenotypes for immunotherapy response and prognosis. Mol Ther Oncol. 2024;32:200890. 39498358 10.1016/j.omton.2024.200890 PMC11532917

[43] Tokuda K Morine Y Miyazaki K. The interaction between cancer associated fibroblasts and tumor associated macrophages via the osteopontin pathway in the tumor microenvironment of hepatocellular carcinoma. Oncotarget. 2021;12:333–43. 33659044 10.18632/oncotarget.27881 PMC7899554

[44] Garnelo M Tan A Her Z. Interaction between tumour-infiltrating B cells and T cells controls the progression of hepatocellular carcinoma. Gut. 2017;66:342–51. 26669617 10.1136/gutjnl-2015-310814 PMC5284473

[45] Xiong YQ Sun HC Zhang W. Human hepatocellular carcinoma tumor-derived endothelial cells manifest increased angiogenesis capability and drug resistance compared with normal endothelial cells. Clin Cancer Res. 2009;15:4838–46. 19638466 10.1158/1078-0432.CCR-08-2780

[46] Li T Zhang X Lv Z Gao L Yan H. Increased expression of myeloid-derived suppressor cells in patients with HBV-related hepatocellular carcinoma. Biomed Res Int. 2020;2020:6527192. 32258134 10.1155/2020/6527192 PMC7097855

[47] Peng Q Shi X Li D. SCML2 contributes to tumor cell resistance to DNA damage through regulating p53 and CHK1 stability. Cell Death Differ. 2023;30:1849–67. 37353627 10.1038/s41418-023-01184-3 PMC10307790

[48] Hussain SP Schwank J Staib F Wang XW Harris CC. TP53 mutations and hepatocellular carcinoma: insights into the etiology and pathogenesis of liver cancer. Oncogene. 2007;26:2166–76. 17401425 10.1038/sj.onc.1210279

[49] Woo HG Wang XW Budhu A. Association of TP53 mutations with stem cell-like gene expression and survival of patients with hepatocellular carcinoma. Gastroenterology. 2011;140:1063–70. 21094160 10.1053/j.gastro.2010.11.034 PMC3057345

[50] Ye S Zhao XY Hu XG. TP53 and RET may serve as biomarkers of prognostic evaluation and targeted therapy in hepatocellular carcinoma. Oncol Rep. 2017;37:2215–26. 28350084 10.3892/or.2017.5494 PMC5367355

[51] Honda K Sbisà E Tullo A. p53 mutation is a poor prognostic indicator for survival in patients with hepatocellular carcinoma undergoing surgical tumour ablation. Br J Cancer. 1998;77:776–82. 9514057 10.1038/bjc.1998.126 PMC2149958

[52] Yano M Hamatani K Eguchi H. Prognosis in patients with hepatocellular carcinoma correlates to mutations of p53 and/or hMSH2 genes. Eur J Cancer. 2007;43:1092–100. 17350822 10.1016/j.ejca.2007.01.032

[53] Park NH Chung YH Youn KH. Close correlation of p53 mutation to microvascular invasion in hepatocellular carcinoma. J Clin Gastroenterol. 2001;33:397–401. 11606857 10.1097/00004836-200111000-00011

[54] Terris B Laurent-Puig P Belghitti J Degott C Hénin D Fléjou JF. Prognostic influence of clinicopathologic features, DNA-ploidy, CD44H and p53 expression in a large series of resected hepatocellular carcinoma in France. Int J Cancer. 1997;74:614–9. 9421358 10.1002/(sici)1097-0215(19971219)74:6<614::aid-ijc10>3.0.co;2-5

