# Supplementary material for: Construction of a prediction model for hepatocellular carcinoma based on machine learning and its prognostic characteristics
Source: Medicine (Baltimore). 2025 Oct 10;104(41):e44966. doi: 10.1097/MD.0000000000044966 (PMC12517959; doi:10.1097/MD.0000000000044966)

Supplementary Figure 1. Data preprocessing for TCGA database and GTEx database. (A) TCGA sample clustering and outlier partitioning. (B) GTEx sample clustering and outlier splitting. (C) PCA analysis of TCGA samples before outlier partitioning. (D) PCA analysis of TCGA samples after outlier partitioning. (E) PCA analysis of GTEx samples before outlier segmentation. (F) PCA analysis of GTEx samples after outlier segmentation.

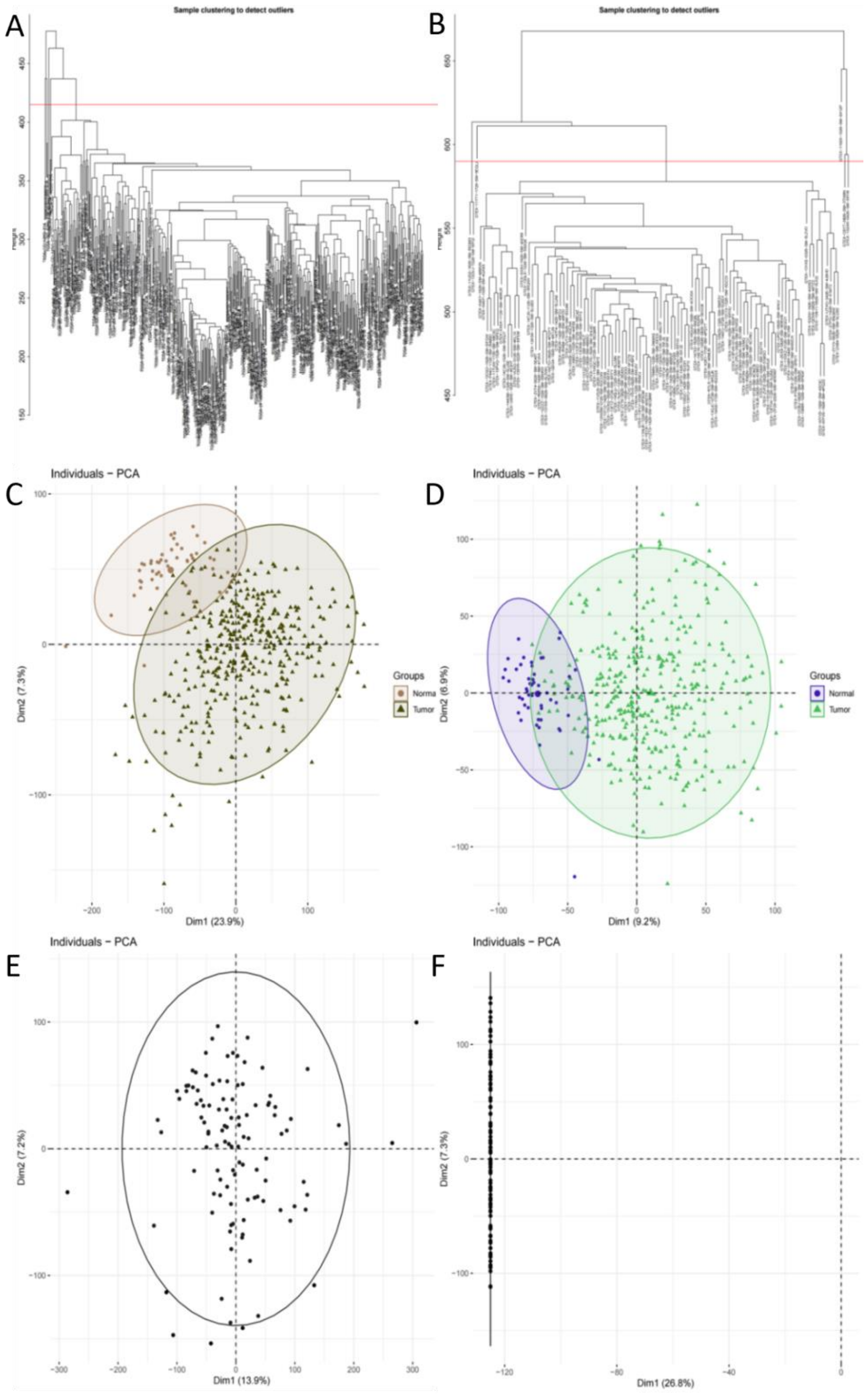

Supplementary Figure 2. Perform preprocessing of combined TCGA database and GTEx database data. (A) PCA analysis of TCGA_GTEx merged data before de-batching. (B) PCA analysis of TCGA_GTEx merged data after de-batching. (C) PCA analysis of TCGA_GTEx subgroups (Normal and Tumor groups) before de-batching. (D) PCA analysis of TCGA_GTEx subgroups (Normal and Tumor groups) after de-batching. (E) TCGA_GTEx box plot before de-batching. (F) TCGA_GTEx boxplot after de-batching.

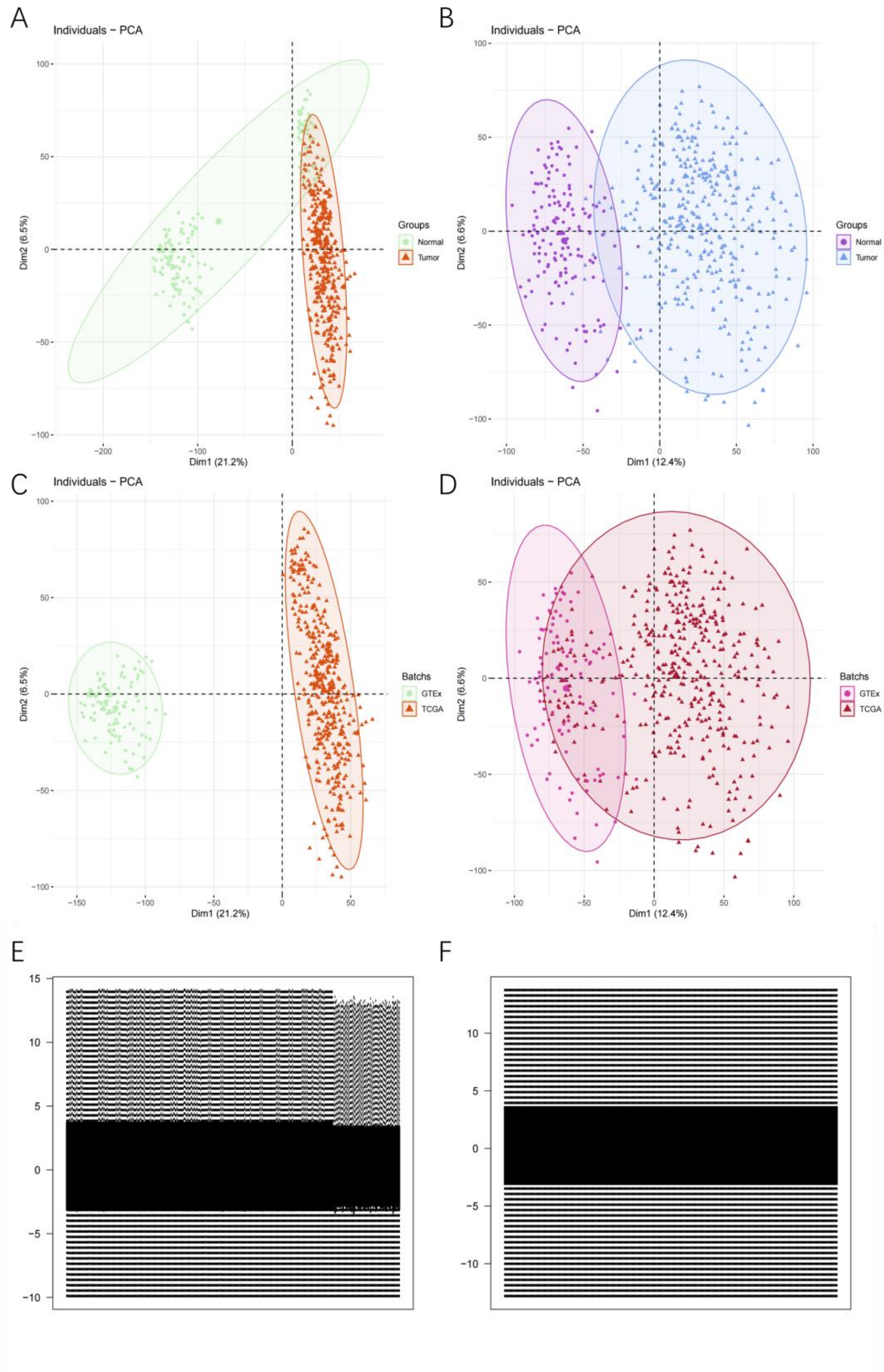

Supplementary Figure 3. K-M survival curves for StepCox[forward]+RSF and RSF models (high- and low-risk groups)

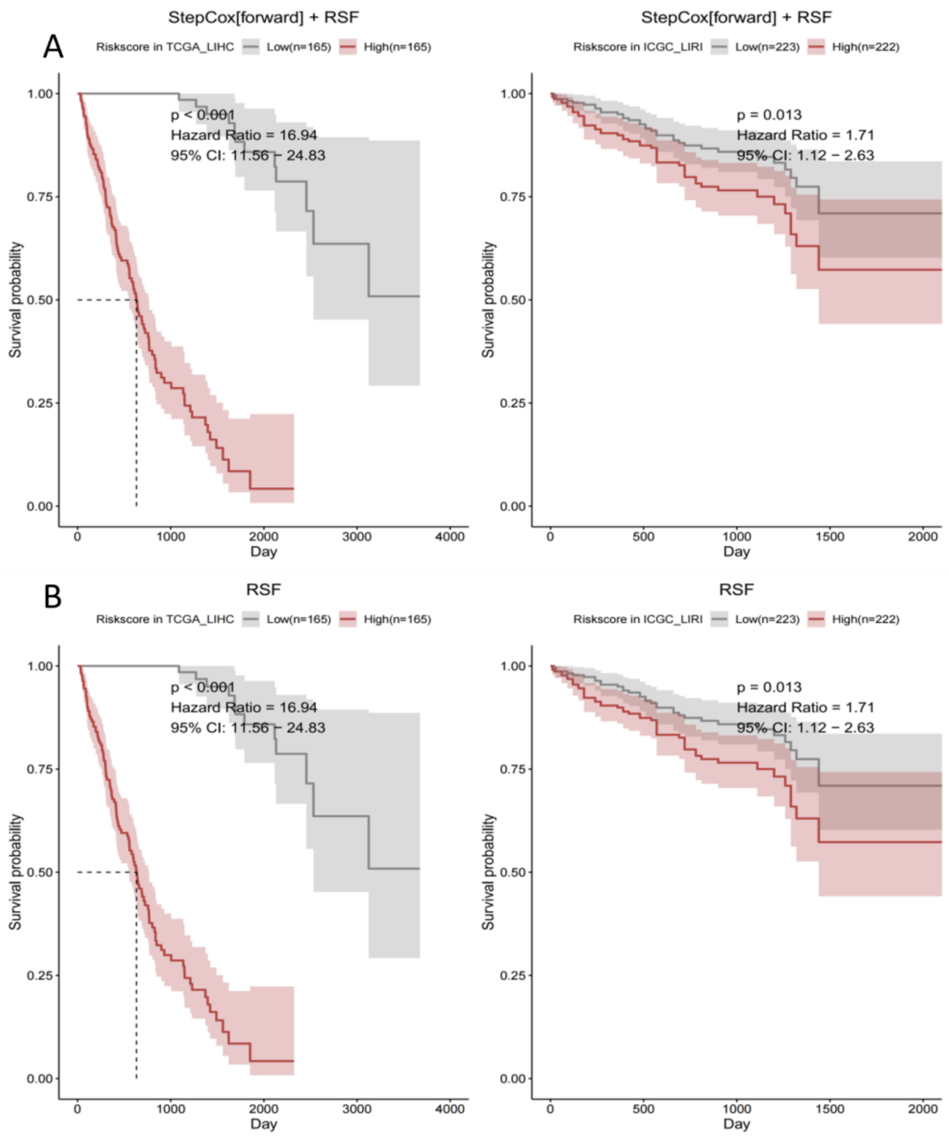

Supplement: Supplementary file 1 [file medi-104-e44966-s001.pdf]
